# Iron-saturated bovine lactoferrin preserves microbiota diversity and healthy ageing-associated taxa in an *in vitro* colon model of elderly gut microbiota (Iron-saturated bovine lactoferrin impact on elderly gut microbiota)

**DOI:** 10.1371/journal.pone.0332631

**Published:** 2025-09-17

**Authors:** María Ruiz-Rico, Huimin Ye, Tom F. O’Callaghan, Paul W. O’Toole, Elaine K. McCarthy

**Affiliations:** 1 School of Microbiology, University College Cork, Cork, Ireland; 2 APC Microbiome Ireland, University College Cork, Cork, Ireland; 3 School of Food and Nutritional Sciences, University College Cork, Cork, Ireland; Wageningen Universiteit, NETHERLANDS, KINGDOM OF THE

## Abstract

Lactoferrin is a multifunctional milk protein receiving increased interest in recent years because of its potential as a functional food ingredient to reduce the prevalence of iron deficiency, while providing additional health benefits including gut microbiota modulation. Iron deficiency represents one of the most prevalent micronutrient deficiencies globally. As such, the aims of this study were (1) to assess the effect of lactoferrin, with different states of iron saturation, on the growth of a simple consortium of gut bacteria, and (2) to evaluate the impact of iron-saturated lactoferrin on the whole gut microbiome of elderly (healthy and frail) donors in an *in vitro* colon model. We report that iron-depleted and native lactoferrin inhibited consortium growth, while the iron-saturated form resulted in changes in consortium composition by enhancing the growth of *Alistipes putredinis.* Fermentation experiments with whole faecal microbiomes showed that the elderly microbiota composition was modified by iron-saturated lactoferrin, mainly in the case of healthy subjects, by altering beta diversity through the modulation of healthy-aging associated taxa including *Coprococcus*, *Alistipes* and *Bifidobacterium*. These findings indicate the positive role that iron saturated lactoferrin may provide in modulating the microbiota of the elderly *in vitro*, increasing the *α*-diversity with the modulation of groups that are normally abundant in healthy individuals and that are typically lost in the transition from a healthy to frail microbiota profile.

## Introduction

The global prevalence of iron deficiency and anaemia at different stages of life is a serious problem. Given their increased iron demands, women and young children are at the greatest risk of iron deficiency [1,2]. Although, the prevalence of iron deficiency in older people ranges from 8–25% in Western countries, which has been shown to increase with age and can rise to 50% in patients in long-term care facilities [3]. Furthermore with an increasing ageing population demographic, this represents an increasing opportunity to develop novel functional foods and ingredients. Iron deficiency in this population is often due to chronic gastrointestinal diseases, which in turn lead to iron loss and malabsorption issues [4]. Iron deficiency can significantly impact quality of life, being associated with fatigue, cognitive dysfunction, decreased muscle strength, and frailty [5].

Traditional approaches to treat iron deficiency have focused on oral iron supplementation, but this strategy has significant limitations. Poor compliance or slow responses to oral ferrous salt preparations can eventually evolve into requiring high-dose supplementation that results in gastrointestinal discomfort [6]. In addition, unabsorbed iron in the gut may subsequently become available as an essential nutrient for siderophilic potential pathogens in the gut resulting in harmful alterations in the gut microbiota [7]. The development of novel strategies to tackle this deficiency is therefore required. Food-based approaches, including food production and fortification, are considered more sustainable and effective strategies for reducing the risk of micronutrient deficiencies, including iron deficiency [8]. Mineral iron fortification can cause technological issues and requires a balanced approach to ensure adequate iron absorption while minimising the impact on food quality and acceptability [8]. As an alternative, food-to-food-fortification consists of the use of natively iron-rich foods or ingredients as fortifiers with potential additional synergistic effects. The whey protein lactoferrin (LF) could represent an ideal high-value ingredient for novel functional food development due to its iron-binding, anti-inflammatory, antimicrobial, immunomodulatory and antioxidant properties [9]. Given these features, LF could offer an interesting dual-purpose potential of providing dietary iron coupled with additional benefits for health [10].

The oral delivery of LF results in higher iron bioavailability in the gastrointestinal tract than ferrous salt preparations [8]. Furthermore, LF can be classified depending on its iron saturation status into the iron-free state (“apo” form), native state (∼15% iron saturation), or iron-saturated state (“holo” form) [11]. The holo form is more resistant to denaturation and proteolysis during food processing and digestion, which can improve its oral bioavailability [12]. The iron saturation status also affects other bioactive properties of LF such as the antimicrobial properties, due to the iron scavenging or release of iron as an essential growth factor, although other antibacterial effects have been reported indicating that LF may exert its effects through iron-independent mechanisms [13,14].

Another potential beneficial effect of LF is the modulation of the gut microbiota by promoting or eliminating specific functionalities or taxa, which may promote host health [15–17]. The gut microbiota of unhealthy aging is characterized by lower diversity, reduced abundance of some subdominant taxa, and the loss of bifidobacterial and fibre-responsive taxa [18]. This has in turn been associated with low-grade inflammation, loss of the gut barrier function and increased susceptibility to pathobiont infections [19,20].

Our research group previously identified a group of taxa whose loss was associated with multiple pathological changes in the elderly and was distinct from that of healthy young/middle-aged individuals [21]. A subset of these taxa was associated with increased frailty (i.e., the aging-related condition characterized by cumulative decline which reduces physiological reserves leading to an increased vulnerability to stressors [22]) in subjects in the ELDERMET cohort (aged 60+) [23]. Indeed, we identified a group of frailty-related taxa present in higher abundance in long-stay-dwelling subjects (referred to as frail) compared to community-dwelling subjects (referred to as healthy) in this cohort. Furthermore, a previous study has linked specific taxa that are positively associated with lower frailty and better cognitive function in the elderly following a Mediterranean diet intervention [24]. Combining the results of these previous studies has enabled us to propose a candidate group of species associated with healthy aging, namely *Alistipes putredinis, Barnesiella intestinihominis, Coprococcus catus, Dorea longicatena, Agathobacter rectalis, Faecalibacterium prausnitzii*, and *Roseburia hominis* (referred to as the S7 consortium) as potential bacteriotherapeutics to promote healthy aging [25]. The potential of this consortium in combination with prebiotics to modulate the gut microbiome of the elderly has been recently investigated by us [26] where we identified enhanced abundance of the species from the synthetic consortium and increased production of beneficial metabolites like short-chain fatty acids.

This consortium can also be used as a reductionist model to study the impact of bioactive compounds, such as LF, on the structure and function of microbial communities in a controlled experimental setting. At a more complex level, *in vitro* whole microbiota models resembling *in vivo* distal gut conditions have been developed to study changes in the microbiota in response to different environmental stimuli [27]. Bioreactors allow simulation of different environmental conditions or host-associated factors and easy monitoring of the ecosystem for characterization by molecular methods such as 16S rRNA gene sequence profiling [28]. These *in vitro* models are ideal systems to assess xenobiotic-induced microbial disturbances and the biochemical profile of diet-microbiota relationship under tightly controlled conditions, with low cost and limited ethical constrains [27]. Supplementation of healthy and frail elderly microbiota types with potential probiotics [29], prebiotics [30] or their combination [26] using an *in vitro* colon model has been previously performed in our research group by measuring effects on the diversity and composition of the microbiota. Thus, the aims of this work were (1) to assess changes in the assembly and growth of the S7 consortium following exposure to different forms of iron-saturated LF and (2) to explore the impact of iron-saturated LF on the faecal microbiota of healthy and frail elderly subjects in a 24-hour *in vitro* batch fermentation system.

## Materials and methods

This study was conducted in two different stages to characterise the impact of LF on the elderly gut microbiota. First, the effect of iron saturation levels of LF (iron-depleted, native and iron-saturated forms) on a consortium of enteric species identified as healthy aging-associated taxa (the so-called S7 consortium) was evaluated by monitoring their impact on the growth kinetics of the consortium and individual taxa in batch culture. The most promising form of LF was then chosen to assess the effect of LF supplementation on the composition of the microbiota from faecal samples of healthy and frail elderly using an *in vitro* colon model.

### LF preparation

Depleted and saturated iron forms were prepared from commercial native LF (Glanbia Nutritionals, IL, USA) for comparison purposes. For the apo LF (iron-depleted form, **LF-A**), 50 mg/mL native protein (**LF-N**) was dissolved in water and was dialyzed against 100 mM citrate buffer with pH 3.5 for 24 h (with three buffer changes), followed by dialysis against distilled water for 24 h with similar water replacement. Holo LF (iron-saturated form, **LF-H**) was prepared by the reaction of 50 mg/mL **LF-N** solution in 50 mM Tris–HCl, 150 mM NaCl, 25 mM sodium bicarbonate (pH 7.4) with ferric nitrate salt over LF in the presence of a weak chelating agent nitrilotriacetic acid (NTA) with the molar ratio of 1:8:8 of lactoferrin:ferric ions:NTA. After 1 h of incubation at room temperature, excess iron was removed by dialysis against water for 24 h (with three water changes). The prepared LF forms were then freeze-dried and stored at room temperature prior to experimental usage. Iron saturation level was estimated based on the A280/A466 ratio according to method developed by Majka et al. [31]. Following this quantification method, the iron saturation level in the native LF (**LF-N**) was 10.0 ± 0.3%, prepared **LF-A** was 2.5 ± 1.8% iron saturation, while **LF-H** iron saturation level was 66.4 ± 5.2%.

### S7 consortium

The S7 consortium comprises seven bacterial species from the previously isolated and characterized Microbiome Culture Collection 100 (MCC100) [29]. The S7 is composed of *Alistipes putredinis, Barnesiella intestinihominis, Coprococcus catus, Dorea longicatena, Eubacterium rectale* (reclassified as *Agathobacter rectalis)* [32], *Faecalibacterium prausnitzii*, and *Roseburia hominis* (see S1 Table for details). S7 species were cultivated in batch in a modified YCFA medium (see S2 Table for detailed composition). All strains were grown in solid and liquid medium at 37°C in strictly anaerobic conditions and were maintained as glycerol stocks at −80°C for long periods. For inoculum preparation, the S7 strains were individually grown in 5 mL of YCFA broth from a single colony and after 48 h, the OD_600_ was measured, and the inoculum was adjusted to an OD_600_ of 0.1 in an anaerobic atmosphere.

### Microbial susceptibility assays

The susceptibility of the bacterial isolates individually, and as part of the S7 consortium, to the different forms of LF was tested using a range of concentrations between 0.5 and 50 mg/mL, which were chosen based on previous studies [33,34].

Bacterial growth was monitored using a 96-well plate. Microplate wells were filled with the required volume of YCFA broth and LF stock solution (50 mg/mL) to obtain 180 µL volumes in each well with the target concentrations of LF. Then, 20 µL of the adjusted inoculum was added to each of the wells and the microplate was incubated anaerobically at 37°C in a plate reader (Cerillo, VA, USA). Growth measurements (OD_600_) were automatically recorded every 30 min over a 24 h-period. For each experiment, control inoculated wells without LF were included to monitor the growth of the strains or consortium in the absence of treatment. All experiments were carried out in quadruplicate. GraphPad Prism (v8) was used to obtain growth kinetic values after baseline correction and to visualise the growth curves.

To investigate the consortium composition after experimental fermentations, total community DNA was subjected to quantitative PCR (qPCR) using primers specifically designed to target the individual S7 16S rRNA gene (see S3 Table for details), according to Ye et al. [25]. At the end of the incubation period, the content of the wells was collected in a microtube and subjected to centrifugation at 16,000 x g for 5 min to separate the cell pellet from the supernatant. Total genomic DNA was obtained with the QIamp Fast DNA Stool kit (Qiagen) following the manufacturer’s instructions. The PCR (total volume of 15 μL) contained 0.75 μL of primers (10 μM), 7.5 μL SYBR Green I, 5 μL nuclease-free water, and 100 ng DNA template. PCR conditions were 95°C for 10 min, followed by 40 cycles of 95°C for 10 s, 55°C for 15 s, and 72°C for 15 s. Fluorescence signals were obtained at the end of each cycle. Amplification and detection were performed using the LightCycler 480 system (Roche, US). All qPCR assays were performed in triplicate. The number of copies/mL was estimated according to standard curves of each strain according to measured cycle threshold values [25]. Log copy number fold changes of each S7 strain were calculated as the difference between the treatment and the control without LF.

### Faecal sample collection

The “healthy” (H2, H3) and “frail” (R7, R11) donors were selected from a subset of the well-phenotyped ELDERMET subjects who live in community-dwelling or long-stay residential care, and whose gut microbiota conformed to the typical clustering patterns correlated to health or frailty in the ELDERMET cohort [23]. The Rockwood Clinical Frailty Scale rating was used to determine their degree of frailty [35]. Donor recruitment (01/04/2021–31/07/2024) was based on exclusion criteria previously described [23], approved by the Clinical Research Ethics Committee of the Cork Teaching Hospitals (study number APC134) and written informed consent was obtained. Upon collection, faecal samples disposed in an air-tight container with an anaerobic generating sachet were transferred to an anaerobic cabinet less than 1 h after defecation. Faecal slurries of 10% w/v were prepared from each homogenized faecal sample in reduced phosphate buffer saline (PBS) and 20% glycerol and then stored in aliquots at −80°C.

### *In vitro* colon fermentation model

The human gut microbiome model was simulated in a single-stage continuous fermentation system mimicking the human colon (MiniBio Reactors, Applikon Biotechnology) as described by Perez et al. [29]. Cultivations were performed in 150 mL working volume with fermentation medium described in S4 Table. The fermentation system was run under the following conditions to mimic the physiological conditions found within the human colon: (i) 37°C, (ii) pH 6.8, (iii) stirring at 80 rpm, and anaerobic conditions through bubbling N_2_ over a period of 24 h according to previous studies of prebiotic impact on faecal microbiota [30,36]. The vessels were kept at pH 6.8 ± 0.1 by the automatic addition of acid (HCl 1 M) or alkali (NaOH 1 M). **LF-H** was added to the sterilized medium at a final concentration of 5 mg/mL. We tested a **LF-H** concentration of 5 mg/mL in line with previous randomized control trial data demonstrating its efficacy in significantly reducing the occurrence of antibiotic-associated diarrhoea in long-term care patients compared with placebo [37]. Faecal aliquots were used as initial inoculum for the fermentation system. Faecal samples were brought from −80ºC to room temperature to thaw under anaerobic conditions before being added to the cultivation vessels. For each donor, four parallel bioreactors were inoculated with the same faecal sample (1% w/v), with two receiving **LF-H** treatment, and two serving as untreated controls. This setup was applied to each of the four donors (two healthy and two frail donors), resulting in a total of 16 bioreactors (n = 8 per group) [38,39]. Samples were collected at 0 h (in the first 5 min after supplementation and inoculation) and at 24 h. Samples were centrifuged at 4500 rpm for 30 min, and pellets and supernatants were kept at −20°C for further analysis. Each experiment was performed in quadruplicate for each faecal sample.

### High-throughput 16S rRNA gene amplicon sequencing and microbiota profiling

DNA was obtained with the QIamp Fast DNA Stool kit (Qiagen). Sequencing of the V3-V4 region of the 16S rRNA gene was performed on an Illumina MiSeq Platform (2 × 250 bp reads) by the Eurofins Genomics Next-Generation Sequencing service (London, UK), using the primer pair CCTAYGGGRBGCASCAG (forward) and GGACTACNNGGGTATCTAAT (reverse). This degenerate version of the widely used 341F/806R primer pair was chosen for its broad coverage of bacterial taxa and reduced amplification bias [38–40]. Sequencing reads were processed using the DADA2 package (version 1.14.0) [41], and produced amplicon sequence variants (ASVs) were classified using SILVA 138.1 database [42]. To achieve species-level taxonomic classification of ASVs, we employed SPINGO, a tool specifically designed for accurate assignment for 16S rRNA gene sequences [43]. Sequencing data were analysed using the software packages *phyloseq* (version 1.48.0) [44] and *microeco* (version 1.7.1) [45] in R (version 4.4.0). For alpha- and beta-diversity analyses, the ASV tables were rarefied at the depth of the smallest library size. To test for statistically significant differences in community composition between groups, we performed Analysis of Similarities (ANOSIM) using the anosim() function from the vegan R package [46]. To identify taxa that were differentially abundant between experimental groups, we used DESeq2 [47], which applies a negative binomial generalized linear model to raw count data.

## Results

### Diverse impact of iron-saturation forms of LF on S7 consortium proportions

**Fig 1:**
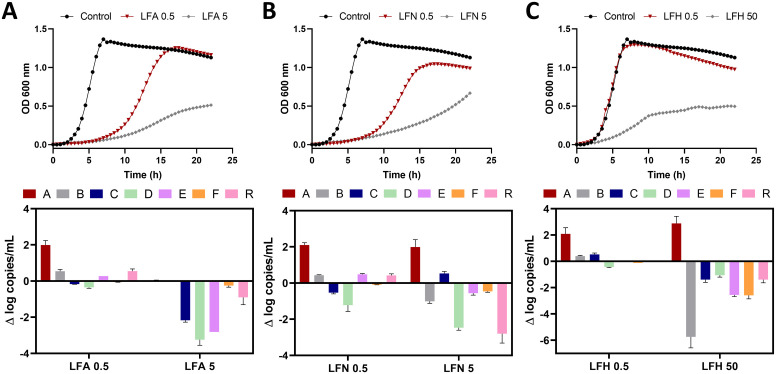
Impact of lactoferrin on the growth of the S7 consortium. Growth curves of the S7 consortium and difference (Δ) in the log of 16S rRNA gene copy number of S7 members after incubation with (A) apo (**LF-A**), (B) native (**LF-N**) and (C) holo (**LF-H**) lactoferrin forms for a 24 h-period. The growth curves values are baseline corrected and representing mean values of four replicates. The log change is relative to that of the S7 without LF and is represented as the ∆ log copies/mL. Concentrations of **LF-A** of 0.5 and 5 mg/mL, **LF-N** of 0.5 and 5 mg/mL, and **LF-H** of 0.5 and 50 mg/mL. Strains are indicated by their genus capital letter as follows: A, *Alistipes putredinis, B, Barnesiella intestinihominis, C, Coprococcus catus, D, Dorea longicatena, E, Eubacterium rectale* (reclassified as *Agathobacter rectalis*)*, F, Faecalibacterium prausnitzii*, and R, *Roseburia hominis.*

For the first stage of evaluating the impact of LF on human gut microbes, the S7 consortium in S3 Table composed of seven culturable and sequenced enteric species identified as healthy aging-associated taxa was chosen [25]. The impact of the different iron-saturation forms of LF on the growth of the S7 consortium is shown in Fig 1. This figure represents the consortium growth curve for two concentrations of the LF forms with a mild or moderate growth effect, compared to the control condition, and the difference (Δ) in the logarithm of the 16S rRNA copy number of the S7 members after 24 h of batch culture. When grown as a consortium, each member strain reached reproducible levels after 24 h of incubation. According to qPCR results the highest copy number was achieved by *D. longicatena* (relative abundance (RA) of 62 ± 10%) followed by *Ag. rectalis* (RA of 12 ± 3%)*, B. intestinihominis* (RA of 11 ± 6%)*, F. prausnitzii* (RA of 9 ± 4%)*, C. catus* (RA of 2 ± 1%)*, R. hominis* (RA of 2 ± 1%) and finally *A. putredinis* (RA 0.9 ± 0.5%)

The LF treatment resulted in a different impact on S7 assembly depending on the iron saturation level (Fig 1). **LF-A** and **LF-N** showed bactericidal effects at the highest concentration (50 mg/mL) and concentrations between 0.5 and 25 mg/mL resulted in a bacteriostatic effect for the S7 consortium. In contrast, the incubation of the S7 culture in the presence of **LF-H** slightly affected the growth of the consortium only at the concentration of 50 mg/mL. Therefore, the effect of LF in the S7 consortium was quantified for two concentrations of the LF forms with a mild or moderate growth effect, The quantification of 16S rRNA gene copies/mL of S7 members in the consortium was performed by qPCR after incubation with different LF forms. Similar to growth curve results, the presence of LF resulted in a decrease of the 16S rRNA gene copies of most of the strains. However, the ability of LF to enhance a member of the consortium was clearly evidenced for *A. putredinis,* especially after incubation with **LF-N** and **LF-H**.

**Fig 2:**
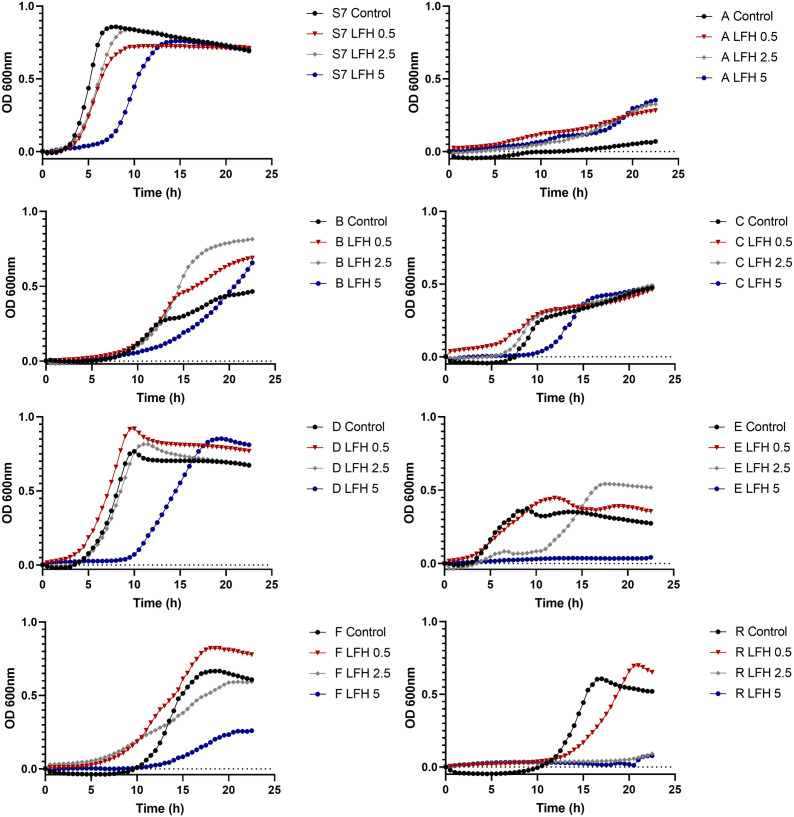
Impact of lactoferrin on the growth of the individual S7 strains and S7 consortium. Growth curves of the individual S7 strains and S7 consortium after incubation with holo lactoferrin (**LF-H**) for a 24 h-period. The growth curves values are baseline corrected and representing mean values of four replicates. Strains are indicated by their genus capital letter as follows: A, *Alistipes putredinis, B, Barnesiella intestinihominis, C, Coprococcus catus, D, Dorea longicatena, E, Eubacterium rectale* (now *Agathobacter rectalis*)*, F, Faecalibacterium prausnitzii*, and R, *Roseburia hominis.*

To test whether the behaviour of the S7 strains was similar growing as part of a consortium compared to growing in pure culture, the impact of **LF-H** on the growth of the consortium and the individually grown strains was studied. Fig 2 shows the growth of individual S7 strains and S7 consortium after incubation with **LF-H** at a range of concentrations between 0.5 and 5 mg/mL (concentrations with a mild effect on growth). The self-assembly of the S7 consortium would be the result of trophic interactions, as can be seen in the different growth curves observed for the consortium grown as a whole or as pure cultures. The S7 consortium was only marginally affected at the concentration of 5 mg/mL, similar to the results shown in Fig 1. In contrast, the influence of **LF-H** on the growth of individual S7 strains was substantially different depending on the strain tested. The growth of *Ag. rectalis* and *R. hominis* was inhibited by high concentrations of **LF-H** showing bactericidal activity. The growth of *C. catus*, *D. longicatena* and *F. prausnitzii* was slightly enhanced by low concentrations of **LF-H,** but higher amounts of **LF-H** resulted in a bacteriostatic effect delaying the growth of the strain or even substantially reducing it. In contrast, the growth of *B. intestinihominis* and, more clearly, *A. putredinis* was enhanced by all **LF-H** conditions tested, which corroborates the results obtained by qPCR with the whole consortium.

### Compositional differences between the faecal microbiota from healthy and frail older donors

Having established that the iron-saturated form of LF (**LF-H**) produced a mild effect in a simple bacterial model, the impact of this LF form on the microbiota of the elderly was tested in an *in vitro* colon model. The system was inoculated with faecal samples from four elderly donors – two healthy individuals living in the community (H2, H3) and two frail subjects in long-stay care (R7, R11). Each sample was run with and without the **LF-H** supplementation (5 mg/mL), and microbiota composition and diversity were studied by 16S rRNA gene sequencing analysis.

First, we confirmed that the faecal microbiota of donors reflected the differences between healthy and frail microbiota types described in previous studies [23,48]. S1 Fig shows how the healthy and frail donor samples used in this study were clustered together with their category members of the ELDERMET cohort based on principal coordinates analysis (PCoA) using Bray–Curtis distances, and statistically significant differences in microbial community composition between groups according to ANOSIM analysis (*p* < 0.01).

**Fig 3:**
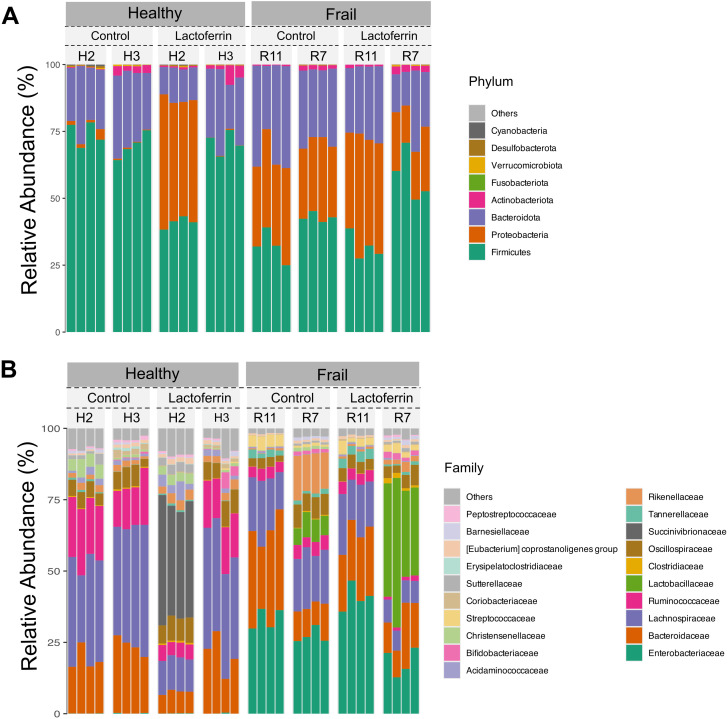
Composition of the healthy and frail microbiota at baseline. Bar graphs of relative abundance at the phylum (A) and family (B) level for the healthy and frail subjects’ microbiota samples with and without the **LF-H** supplementation at time point 0 h. Each column represents a sample.

The composition of the healthy and frail type faecal microbiota at phylum and family level at baseline (time point 0 h) is shown in Fig 3. The composition of the healthy and frail faecal microbiota at genus level at baseline is also shown in S2 Fig. The two types of microbiota showed clear differences, although one of the healthy donors (H2) and one of the frail donors (R7) showed an inconsistent microbiota composition for all samples. The compositional differences between healthy and frail donors include significantly reduced relative abundance of *Lachnospiraceae* (mainly *Agathobacter* and *Blautia* genera) and *Ruminococcaceae* (mainly *Faecalibacterium* genus) in the faecal microbiota of frail subjects compared to healthy. Furthermore, frail donor samples presented significant enrichment in Pseudomonadota (Proteobacteria) abundance including *Enterobacteriaceae* (main genera in the frail samples being *Escherichia-Shigella* and *Klebsiella*).

**Fig 4:**
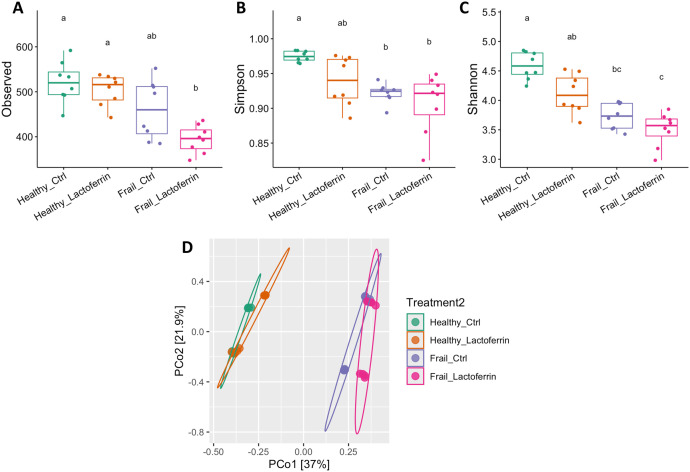
Compositional differences between the faecal microbiota from healthy and frail older donors at time point 0 h. Boxplot of *α*-diversity analysis based on observed ASV (A), Simpson index (B) and Shannon index (C) of healthy and frail microbiota samples with and without the **LF-H** supplementation. Statistical significance was determined for the boxplots using Dunn’s Kruskal-Wallis Multiple Comparisons. Different letters (a, b, c) in the plots indicate statistically significant differences between groups (*p* < 0.05). Principal Component Analysis (PCoA) of *β*-diversity (Bray–Curtis dissimilarity) at the ASV level of the healthy and frail microbiota samples with and without the **LF-H** supplementation (D). Pair-wise comparison *p*-value included in S5 Table.

The differences in faecal microbiota *α*-diversity according to observed ASV, Simpson and Shannon indices of healthy and frail subject microbiota samples at baseline (time point 0 h), are shown in Fig 4A-C. Furthermore, Fig 4D and S3 Fig show PCoA of *β*-diversity based on using Bray–Curtis dissimilarity (considering the taxon abundance) and unweighted UniFrac distance (considering the presence and absence of taxa) to investigate differences in microbiota composition at the ASV level. As expected *α*-diversity analysis showed statistically significant diversity between healthy and frail microbiota samples. However, no effect of the **LF-H** supplementation on microbiota *α*-diversity was detectable at baseline. Based on the PCoA of the Bray–Curtis dissimilarity (Fig 4D) and unweighted UniFrac distance (S3 Fig) in the faecal samples at time 0 h, untreated and treated microbiota samples clustered by donor type. Pair-wise comparison of *β*-diversity showed no differences between untreated and LF-treated for both type of donors at baseline (see S5 Table and S3 Fig). This indicates that **LF-H** did not override the strong subject-specific microbiota signatures, allowing us to assess **LF-H** effects within each donor’s consistent microbial background.

### Holo lactoferrin supplementation significantly affected microbiota composition

**Fig 5:**
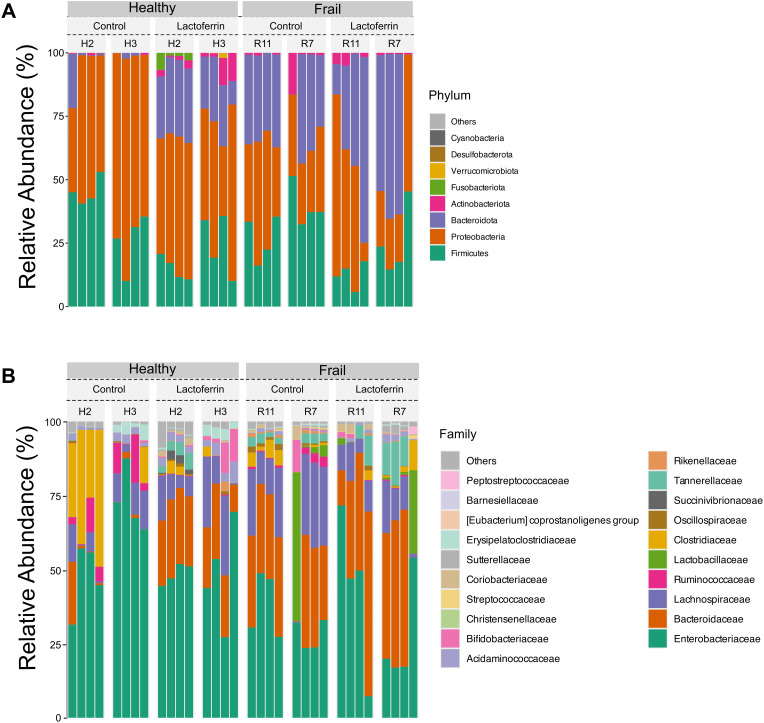
Composition of the healthy and frail microbiota after 24 h-fermentation. Bar graph demonstrating relative abundance at the phylum (A) and family (B) level for the healthy and frail subject microbiota samples with and without the **LF-H** supplementation at time point 24 h.

The microbiota profile at phylum and family level for healthy and frail samples after 24 h-fermentation with **LF-H** is shown in Fig 5. The composition of the healthy and frail faecal microbiota samples at genus level at time point 24 h is also shown in S4 Fig. A significant increase in Pseudomonadota (Proteobacteria) relative abundance was observed after cultivation with basal medium or **LF-H** for both types of groups. This was especially evident for healthy samples after cultivation with basal medium, a high relative abundance was observed of *Enterobacteriaceae* (*Escherichia-Shigella* group, 60%) and *Clostridiaceae* (Clostridium *sensu stricto* 1 group, 18%)*.* For both groups, high Bacteroidota and *Bacteroidaceae* relative abundance was observed after cultivation with **LF-H** (18% in healthy samples and 36% in frail samples). For healthy samples, the high baseline proportion of the health-associated *Lachnospiraceae* was better maintained when the fermentation was supplemented with **LF-H** (7% *vs.* 15% in control and LF-supplemented samples).

**Fig 6:**
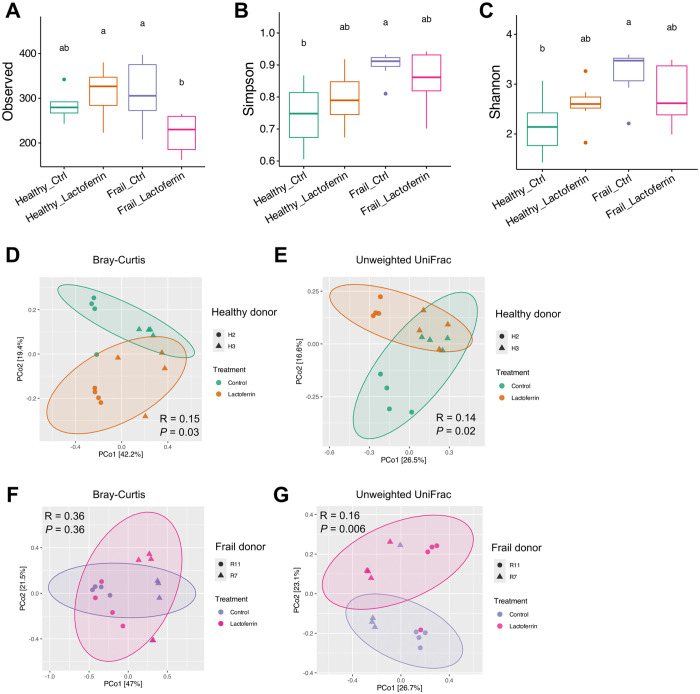
Compositional differences between the faecal microbiota from healthy and frail older donors at time point 24 h. Boxplot of *α*-diversity analysis based on observed ASV (A), Simpson index (B) and Shannon index (C) of healthy and frail microbiota samples with and without the **LF-H** supplementation. Statistical significance was determined for the boxplots using Dunn’s Kruskal-Wallis multiple comparisons. Different letters (a, b, c) in the plots indicate statistically significant differences between groups (*p* < 0.05). Principal Component Analysis (PCoA) of *β*-diversity (Bray–Curtis dissimilarity and Unweighted UniFrac) at the ASV level of the healthy (D, E) and frail (F, G) microbiota samples with and without the **LF-H** supplementation.

Concerning the effect of **LF-H** on the gut microbiota composition, the values of *α*-diversity are presented in Fig 6A-C and PCoA *β*-diversity (Bray–Curtis dissimilarity) are shown in Fig 6D-G. Regardless of supplementation or not of the medium, there was an overall decrease in the observed ASVs of the faecal microbiota after 24 h *in vitro* fermentation compared to baseline (Fig 6A). Supplementation of **LF-H** did not affect the *α*-diversity of healthy microbiota (Fig 6A-C). The **LF-H** supplementation significantly decreased the observed ASVs but did not affect the Simpson and Shannon indices of the frail microbiota (Fig 6A-C). In contrast, **LF-H** supplementation in healthy microbiota significantly altered *β*-diversity compared to the healthy control based on both Bray-Curtis (*p* = 0.03, Fig 6D) and Unweighted UniFrac distances (*p* = 0.02, Fig 6E). No effect was observed in frail microbiota based on Bray-Curtis dissimilarity (Fig 6F, *p* = 0.33); however, a significant difference was detected using unweighted UniFrac distances (Fig 6G*, p* = 0.006). Taking into account that unweighted UniFrac distance measures the phylogenetic dissimilarity of samples by estimated taxa presence/absence, the significant difference for this beta-diversity index may suggest that the differing taxa between frail samples after **LF-H** supplementation are not close phylogenetically.

**Fig 7:**
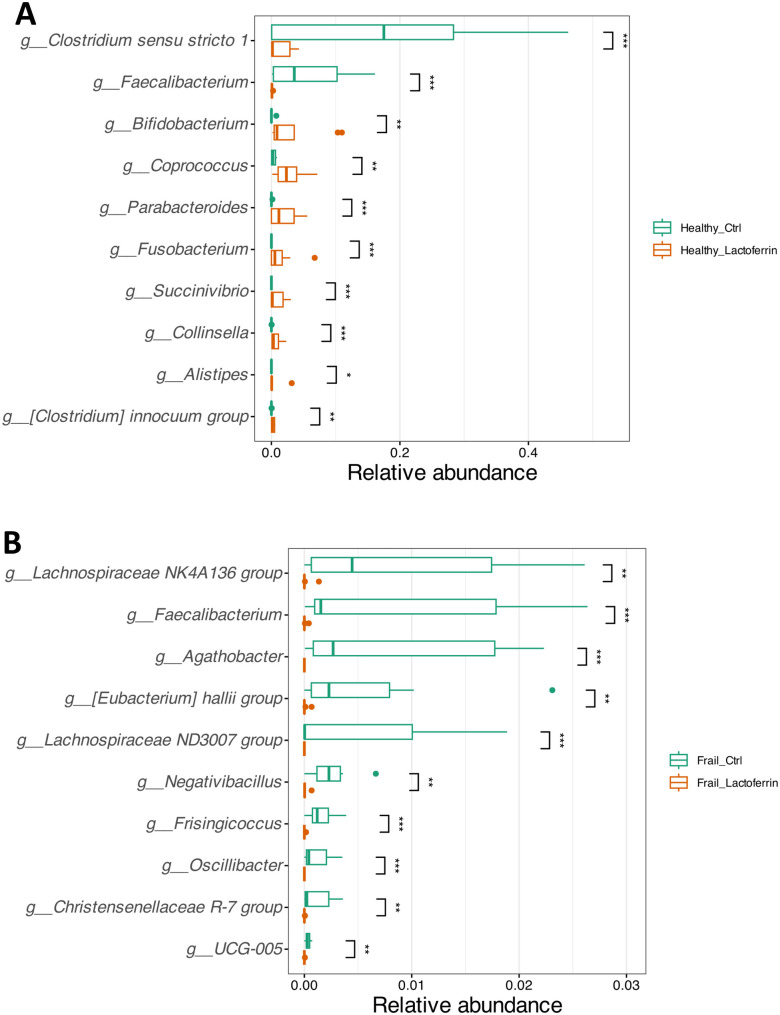
Differential taxa between the untreated and treated healthy and frail samples after 24 h-fermentation. DeSeq2 analysis of differential taxa between the microbiota of healthy (A) and frail (B) donors with and without the **LF-H** supplementation. * p < 0.05, ** p < 0.01, *** p < 0.001.

To provide further resolution of the effect of **LF-H** supplementation, we used DESeq2 to identify differential taxa between the untreated and treated healthy and frail samples after 24 h cultivation (Fig 7). Supplementation with **LF-H** on the cultivation basal medium seeded with either microbiota type decreased the abundance of *Faecalibacterium*. In addition, **LF-H** supplementation resulted in a decrease of *Clostridium sensu stricto 1* group in the healthy microbiota samples and a decrease of *Lachnospiraceae* NK4A136 and ND3007 groups, *Eubacterium hallii* group, *Christensenellaceae* R-7 group, *Agathobacter*, *Negativibacillus*, *Frisingicoccus*, *Oscillibacter* and UCG-005 genera in the frail microbiota samples. In contrast, **LF-H** supplementation resulted in an increase in the abundance of *Bifidobacterium, Coprococcus, Parabacteroides, Fusobacterium, Succinivibrio, Collinsella, Alistipes* and *Clostridium innocuum* group only with microbiota samples from healthy donors.

Lastly, to compare the effect of **LF-H** on the S7 consortium and the *in vitro* colon model, we applied SPINGO to our 16S rRNA amplicon sequencing data to enable taxonomic classification of ASVs to the species level at 24 h (see S5 Fig). *B. intestinihominis* was not detected after 24 h of fermentation. In the microbiota from frail donors, **LF-H** treatment significantly reduced the relative abundances of *C. catus* (C)*, D. longicatena* (D)*, Ag. rectalis* (E) and *F. prausnitzii* (F). Conversely, in the microbiota from healthy donors, **LF-H** increased the relative abundances of species *C. catus* (C)*, D. longicatena* (D)*,* and *R. hominis* (R), while significantly reduced the relative abundance of *F. prausnitzii* (F).

## Discussion

The objective of this study was to characterise the effects of LF iron saturation levels in two microbiota models (S7 consortium and faecal microbiota from elderly subjects). The effect of **LF-A**, **LF-N** and **LF-H** on the assembly and growth of a consortium of enteric species identified as healthy aging-associated taxa (S7 consortium) was different according to the iron saturation level. The composition of the S7 consortium results from a delicate balance of microbial interactions, the growth rates of the individual strains, and the carrying capacity (i.e., maximum population size that can be sustained) of the experimental system [49]. The consortium self-assembly driven by trophic interactions between microbial species is difficult to establish by absorbance and qPCR, but these techniques provide valuable information on how the S7 consortium responds to a specific perturbation in the environment (LF supplementation).

**LF-A** and **LF-N** showed bacteriostatic (i.e., inhibition of bacterial growth) or bactericidal (i.e., killing of bacteria) effects according to their concentration, while **LF-H** only slightly affected the growth of the consortium (Fig 1). **LF-A** and **LF-N** have the ability to sequester environmental iron, thus limiting the growth of some bacteria, as it is an essential element for them [50]. The bactericidal action of LF is also associated with its disruptive effect on osmosis due to its cationic N-terminus that reacts with the anionic lipopolysaccharides in Gram-negative, and lipoteichoic acid in Gram-positive bacterial cell membranes [51]. On the other hand, certain bacteria have developed mechanisms for capturing iron from **LF-H**, using it for their metabolism with potential enhancement of their growth [50]. This type of growth enhancement was observed for *A. putredinis,* especially after incubation with **LF-H** (Fig 1-2), in accordance with previous investigations [52].

Having established the mild effect of **LF-H** on the simple enteric bacterial model S7, the impact of **LF-H** on the microbiota of the elderly was investigated in an *in vitro* colon model using two types of faecal microbiota, subjects who live in community (healthy) and subjects who live in long-stay residential care (frail). The two microbiota types had major compositional differences at the outset, summarized by a reduced overall diversity and abundance of Bacillota, and an enrichment in Pseudomonadota, especially *Enterobacteriaceae,* in the frail type compared to the healthy one (Fig 3). Previous studies have revealed that frail elderly subjects living in long-term residential care units have a lower diversity of a characteristic microbiota composition compared to that of community-dwelling subjects [48]. The faecal microbiome composition of frail donors (Fig 3) is consistent with previous descriptions of the microbiome in elderly subjects [18], allowing us to conclude that the samples used in this study were suitable representatives to study the effect of LF on the microbiota of elderly people.

**LF-H** addition to the cultivation basal medium did not significantly affect the microbiota composition at baseline (Fig 4), but after a 24-h period of fermentation with or without **LF-H** supplementation, a loss of *α*-diversity was observed for both the healthy and frail microbiota samples (Fig 6). This loss of diversity is common of *in vitro* gastrointestinal models, indicating *in vitro* models have limitations in their ability to mimic human colon conditions with eventual collapse of the system [30]. Analysis of the microbiota profiles in healthy and frail samples with and without the **LF-H** addition showed significant differences in composition and diversity at time 24 due to **LF-H** exposure (Fig 5-6). The dominant families in all the samples after cultivation were *Enterobacteriaceae, Bacteroidaceae*, *Lachnospiraceae* and *Clostridiaceae.* However, **LF-H** addition resulted in a dominance of the *Bacteroidaceae* family compared to control samples. 53 et al. (2022) used a murine model of antibiotic-induced dysbiosis to determine the potential of **LF-N** and **LF-H** to reverse the effects on gut microbiota after clindamycin treatment [53]. The authors reported that bacteria from families *Bacteroidaceae*, *Prevotellaceae* and *Rikenellaceae* (belonging to the Bacteroidota phylum) were decreased in antibiotic-treated mice, but the treatment with **LF-N** or **LF-H** along with clindamycin reversed these effects, increasing the levels of the bacteria of all these families. *Bacteroidaceae* family, to which the genus *Bacteroides* belongs, includes important opportunistic pathogens, but as highly saccharolytic members of a typical microbiota dominated at phylum level by Bacteroidota and Bacillota, they are considered a relevant health-maintaining family responsible of producing short chain fatty acids [54].

We identified differential taxa for healthy and frail microbiome type samples according to DESeq2 (Fig 7), with a deeper focus on compositional differences in untreated and LF-treated samples from healthy donors in which the *β*-diversity was significantly altered (Fig 6D-E). **LF-H** supplementation resulted in a decreased abundance of *Faecalibacterium* genus for the healthy and frail type microbiota. These results are in accordance with the results obtained for the S7 consortium (Fig 2) and SPINGO analysis (S5 Fig), in which the growth of the strain *F. prausnitzii* was negatively affected by the presence of **LF-H**. Furthermore, **LF-H** supplementation resulted in a decrease of *Clostridium sensu stricto 1* group in healthy samples, which contain *Clostridium* species generally perceived as pathogenic and indicators of a less healthy microbiota [55]. Likewise, the treatment of a microbiota model infected with *C. difficile* with **LF-H** resulted in a delay of *C. difficile* vegetative cell growth and prevention of toxin production [33]. In addition, this study reported that the presence of excess iron alone did not prevent cell proliferation and toxin production, indicating the multiple mechanisms of action of LF.

**LF-H** treatment also led to an increase in the relative abundance of *Bifidobacterium, Coprococcus* and *Alistipes* genera, among others, but only in healthy elderly donor samples. These results agree with the data obtained for the S7 consortium (Fig 2) and SPINGO analysis (S5 Fig), in which the growth of *A. putredinis* and *C. catus* was enhanced by the presence of **LF-H.** Hu et al. (2020) previously evaluated the effect of LF administration on the gut microbiota of suckling piglets and reported an increase in the relative abundance of genera from Bacteroidota (*Butyricimonas* and *Prevotella*) and Bacillota (*Coprococcus, Roseburia* and *Ruminococcus*) responsible for producing short chain fatty acids accompanied by lower abundance of the opportunistic pathogens *Escherichia/Shigella* group after the LF treatment [16]. The increased abundance of *Bifidobacterium* after LF supplementation has previously been reported in different microbiota types such as infants [56] and obese adults [57]. 57 reported a significant increase in *Bifidobacterium* genus abundance and depleted Enterobacteriales in the gut microbiome in a high-fat diet induced obese model after supplementation with LF [57]. *Bifidobacterium* spp. includes some strains that are well-known probiotics with multiple beneficial effects on intestinal physiology, including modulating gut microbiota, improving the immune system, or enhancing intestinal barrier function. In addition, as the elderly microbiota is characterised by the loss of bifidobacterial taxa [18], the increase in abundance of this taxon indicates potential health benefits deriving from the consumption of the prebiotic LF [34].

Altogether, these findings indicate the positive role of **LF-H** in modulating the microbiota of the elderly *in vitro*, increasing the *α*-diversity with the modulation of groups that are normally abundant in healthy individuals and that are lost in the transition from a healthy to frail microbiota profile.

This study has a number of limitations. The first is inherent to fermenter studies, relating to the number of samples and replicates that can be tested. Given the labour intensiveness and duration of the experiments, it was not possible to assess the effects of multiple doses of LF or different levels of iron-saturation on the gut microbiome model. As a second limitation, faecal samples were treated individually, rather than pooling samples from several sample points or from several donors, an approach used in some other studies. We chose this option because pooling faecal samples produces inocula that may not be representative of the average “real” microbiota, and that contain artificial metabolic redundancies that may obscure the normal trophic interactions [58]. However, in this study we found inconsistent microbiota composition in two of the four donors at baseline, which made it difficult to observe differences in composition and diversity after LF treatment. The use of only two donors per group represents a key limitation, as this small sample size restricts the statistical power and generalizability of our findings. While the inclusion of four parallel bioreactors per donor allowed for technical replication and assessment of treatment effects within individuals, inter-individual variability remains a major factor influencing microbiome responses. In addition, the fermentation medium contains a mixture of prebiotics to prevent the collapse of the system during the 24 h incubation period, although these components may mask the effect of LF on the composition of the microbiota given the positive impact of this prebiotic mix on the ecosystem. A further limitation of our study is the lack of absolute bacterial quantification in the *in vitro* fermentation model, such as quantification of S7 measured by qPCR.

Despite limitations, this study provides valuable preliminary insights on the responses of the microbiota to **LF-H** supplementation in frail and healthy elderly. We tested a concentration of **LF-H** (5 mg/mL) in line with previous studies focusing on humans with gut microbiome dysbiosis [33,37]. Furthermore, the total amount of **LF-H** in the bioreactor (i.e., for a vessel with a working volume of 150 mL, 750 mg of **LF-H**) is in the range of the oral dose of lactoferrin with proven beneficial effects on inflammation and immune function in humans (0.3–3 g/d) [59]. However, further research is needed to assess the stability and function of this form of LF when incorporated into functional foods targeting this population group. Given the desire to adopt more food-first approaches to tackle micronutrient deficiencies at the population level; the impact of the whole food matrix and its effects on subsequent iron bioavailability should also be investigated during any ingredient development with LF [10]. Moreover, the impact of the protein itself on the intestinal barrier, iron absorption and overall effect on host health needs further study. Iron saturation of LF can have significant effects on protein structure, as iron binding results in a more compact tertiary structure of the protein that affects functional properties. Iron-saturated LF is therefore more resistant to thermal denaturation and proteolysis, but the release of iron from the protein depends on destabilisation of the holo form [11]. Thus, the behaviour of iron-saturated LF incorporated into food needs to be characterised throughout the digestion process with a view to creating a more robust functional ingredient. The role of LF-enriched foods in the prevention and treatment of iron deficiency, while potentially preventing or combatting frailty in the elderly merits further investigation through *in vitro* studies and well-designed human intervention trials. The current study adds to a growing evidence base that highlights the potential health-benefiting properties of LF across the population.

## Supporting information

S1 Fig Mapping of donors on the ELDERMET cohort. Beta diversity comparisons based on Bray-curtis distances of the microbiota of community-dwelling and long-stay-dwelling elderly subjects. The microbiota of selected donors (triangles) was compared to that of a subset ELDERMET cohort (circles; CM, n = 79; LS, n = 59). Statistically significant difference in microbial community composition between groups was analyzed using ANOSIM.
(TIF)

S2 Fig Composition of the healthy and frail microbiota at baseline. Histogram of relative abundance at the genus level for the non-treated and lactoferrin-treated microbiota samples at baseline (time 0).
(TIF)

S3 Fig Compositional differences between the faecal microbiota from healthy and frail older donors at baseline (time 0 h). (A) Principal Component Analysis (PCoA) of *β*-diversity based on unweighted UniFrac distance at baseline. (B) Comparisons of unweighted UniFrac distances at baseline between healthy and frail microbiota samples with and without the **LF-H** supplementation (n = 4).
(TIF)

S4 Fig Composition of the healthy and frail microbiota after 24-h fermentation. Histogram of relative abundance at the genus level for the non-treated and lactoferrin-treated microbiota samples at time-point 24 h.
(TIF)

S5 Fig S7 species in healthy and frail microbiota after 24-h fermentation according to SPINGO analysis. Bar graphs of relative abundance of *A. putredinis, C. catus, D. longicatena, E. rectale* (reclassified as *Ag. rectalis*)*, F. prausnitzii* and *R. hominis* for the non-treated and lactoferrin-treated microbiota samples at time-point 24 h. Pair-wise comparison *p*-values between non-treated and lactoferrin-treated microbiota samples (*p* < 0.05).
(TIF)

S1 Table S7 consortium. Taxonomic information and the genome accession number of S7 taxon.
(XLSX)

S2 Table S7 consortium growth media. Composition of YCFA media used to grow S7 consortium in the susceptibility assays (per L of media).
(XLSX)

S3 Table S7 consortium primers. S7 strains specific primers used in this study.
(XLSX)

S4 Table *In vitro* colon media. Composition of the growth medium used *in vitro* colon fermentation experiments (per L of media).
(XLSX)

S5 Table Multivariate analysis of variance based on beta diversity at 0 h. (XLSX)
