# Supplementary figures and images for: Iron-saturated bovine lactoferrin preserves microbiota diversity and healthy ageing-associated taxa in an in vitro colon model of elderly gut microbiota (Iron-saturated bovine lactoferrin impact on elderly gut microbiota)
Source: PLoS One. 2025 Sep 17;20(9):e0332631. doi: 10.1371/journal.pone.0332631 (PMC12443303; doi:10.1371/journal.pone.0332631)

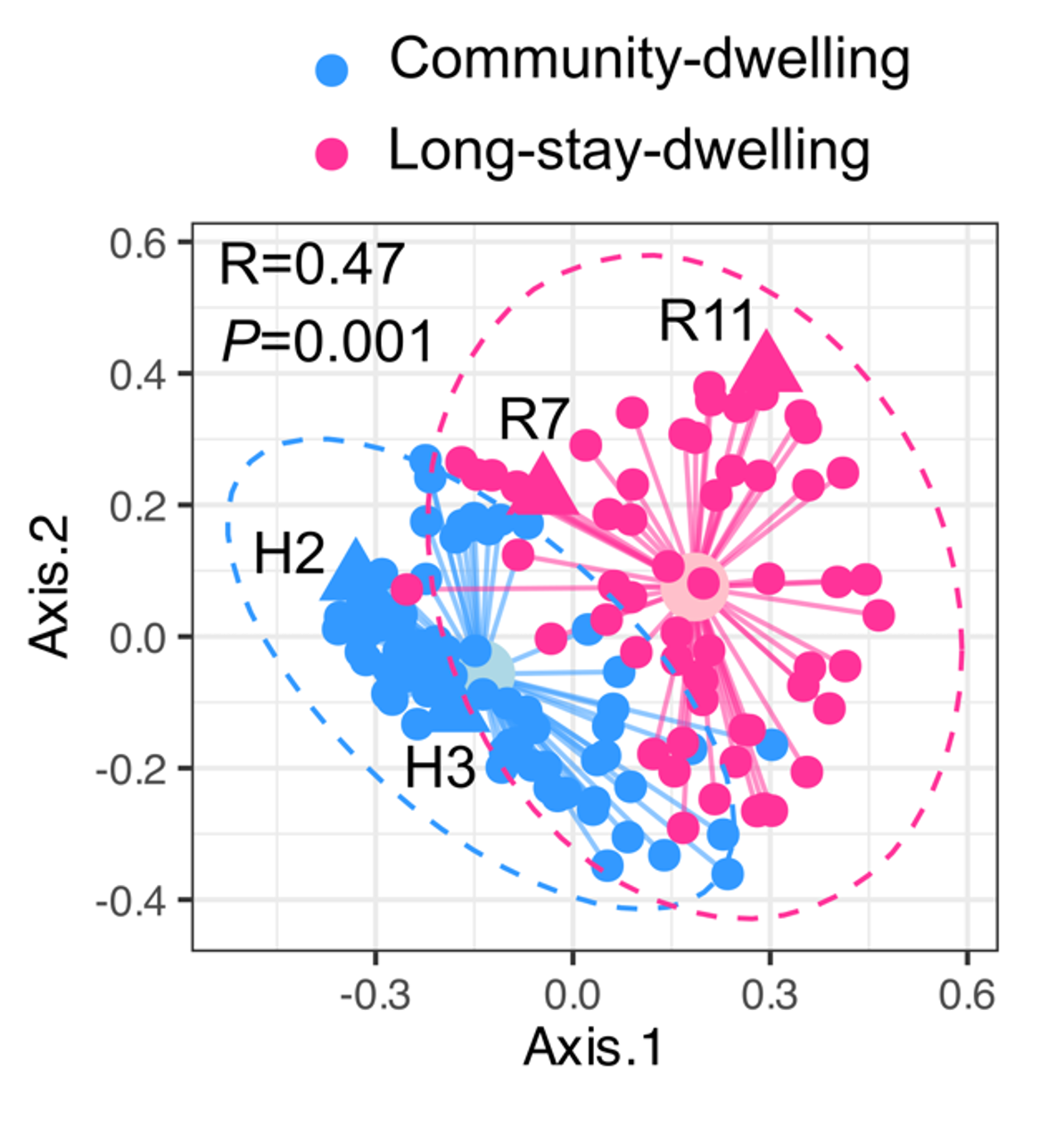

Supplement: S1 Fig — Beta diversity comparisons based on Bray-curtis distances of the microbiota of community-dwelling and long-stay-dwelling elderly subjects. The microbiota of selected donors (triangles) was compared to that of a subset ELDERMET cohort (circles; CM, n = 79; LS, n = 59). Statistically significant difference in microbial community composition between groups was analyzed using ANOSIM. (TIF) [file pone.0332631.s001.tif]

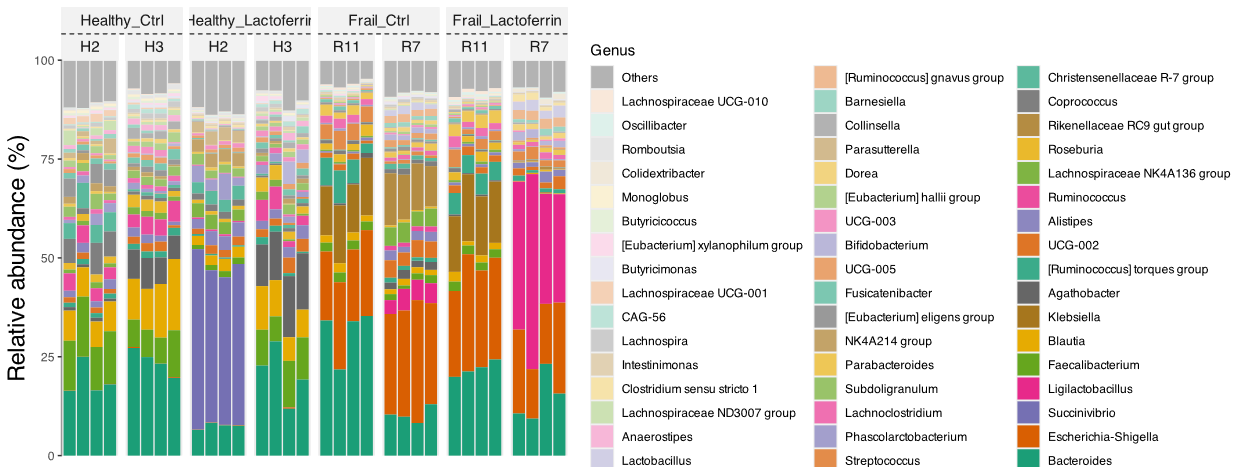

Supplement: S2 Fig — Histogram of relative abundance at the genus level for the non-treated and lactoferrin-treated microbiota samples at baseline (time 0). (TIF) [file pone.0332631.s002.tif]

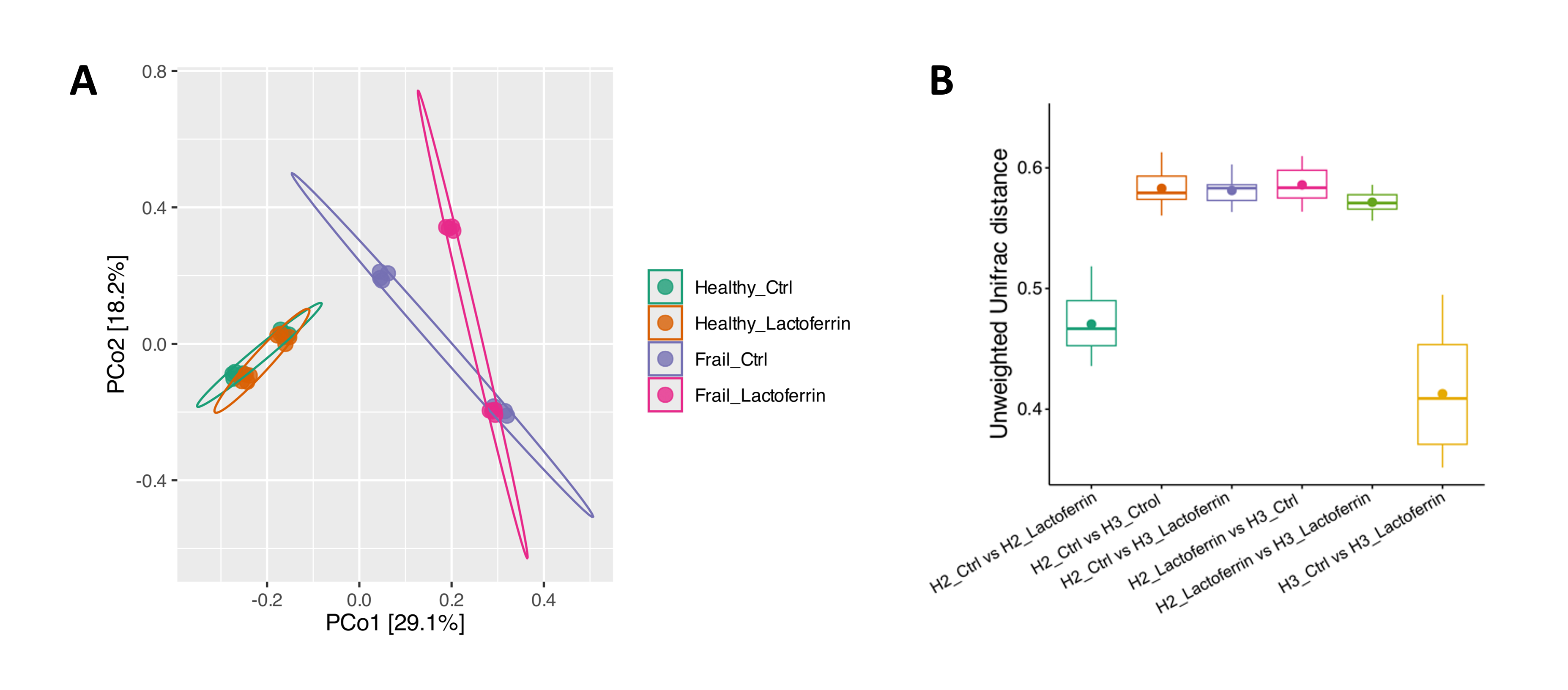

Supplement: S3 Fig — (A) Principal Component Analysis (PCoA) of β-diversity based on unweighted UniFrac distance at baseline. (B) Comparisons of unweighted UniFrac distances at baseline between healthy and frail microbiota samples with and without the LF-H supplementation (n = 4). (TIF) [file pone.0332631.s003.tif]

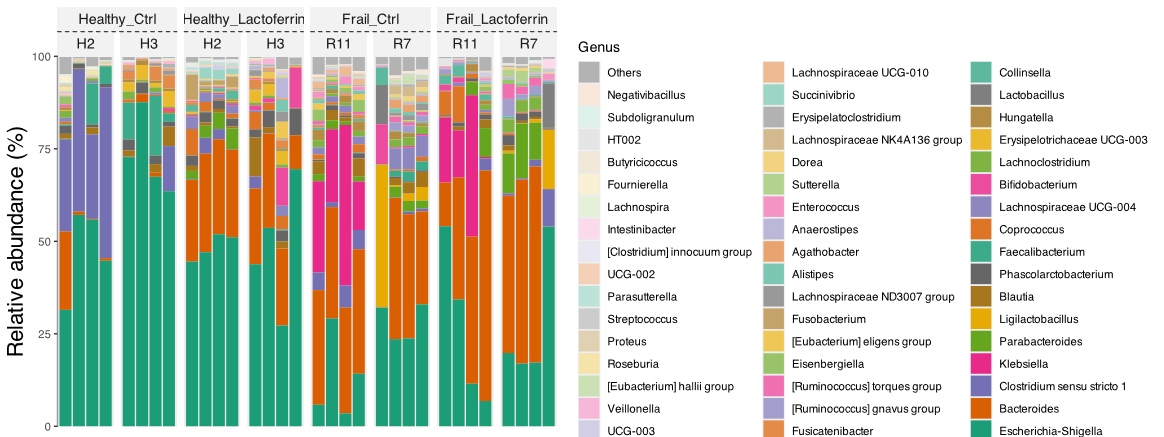

Supplement: S4 Fig — Histogram of relative abundance at the genus level for the non-treated and lactoferrin-treated microbiota samples at time-point 24 h. (TIF) [file pone.0332631.s004.tif]

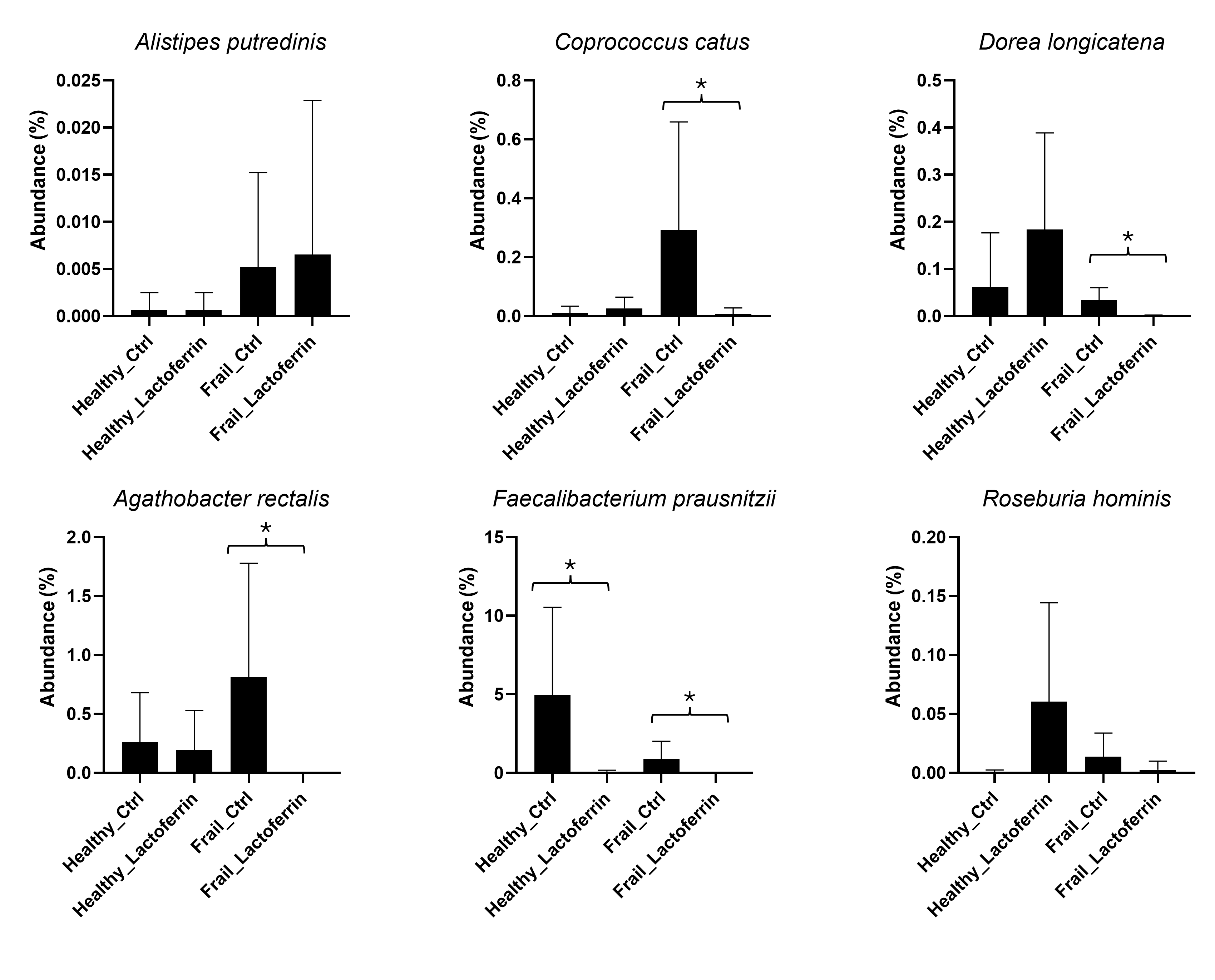

Supplement: S5 Fig — Bar graphs of relative abundance of A. putredinis, C. catus, D. longicatena, E. rectale (reclassified as Ag. rectalis), F. prausnitzii and R. hominis for the non-treated and lactoferrin-treated microbiota samples at time-point 24 h. Pair-wise comparison p-values between non-treated and lactoferrin-treated microbiota samples (p < 0.05). (TIF) [file pone.0332631.s005.tif]
